# Transcriptional alterations in bladder epithelial cells in response to infection with different morphological states of uropathogenic *Escherichia coli*

**DOI:** 10.1038/s41598-021-04396-0

**Published:** 2022-01-11

**Authors:** Katarina Persson, Ulrika Petersson, Charlotte Johansson, Isak Demirel, Robert Kruse

**Affiliations:** 1 Faculty of Medicine and Health, iRiSC - Inflammatory Response and Infection Susceptibility Centre, Örebro University, Örebro, Sweden; 2 School of Medical Sciences, Örebro University, Örebro, Sweden; 3 School of Health Sciences, Örebro University, Örebro, Sweden; 4 Department of Clinical Research Laboratory, Faculty of Medicine and Health, Örebro University, Örebro, Sweden

**Keywords:** Bacterial infection, Urinary tract infection, Immune evasion, Infection, Antimicrobial responses

## Abstract

Uropathogenic *Escherichia coli* (UPEC) may undergo a cyclic cascade of morphological alterations that are believed to enhance the potential of UPEC to evade host responses and re-infect host cell. However, knowledge on the pathogenic potential and host activation properties of UPEC during the morphological switch is limited. Microarray analysis was performed on mRNA isolated from human bladder epithelial cells (HBEP) after exposure to three different morphological states of UPEC (normal coliform, filamentous form and reverted form). Cells stimulated with filamentous bacteria showed the lowest number of significant gene alterations, although the number of enriched gene ontology classes was high suggesting diverse effects on many different classes of host genes. The normal coliform was in general superior in stimulating transcriptional activity in HBEP cells compared to the filamentous and reverted form. Top-scored gene entities activated by all three morphological states included IL17C, TNFAIP6, TNF, IL20, CXCL2, CXCL3, IL6 and CXCL8. The number of significantly changed canonical pathways was lower in HBEP cells stimulated with the reverted form (32 pathways), than in cells stimulated with the coliform (83 pathways) or filamentous bacteria (138 pathways). A host cell invasion assay showed that filamentous bacteria were unable to invade bladder cells, and that the number of intracellular bacteria was markedly lower in cells infected with the reverted form compared to the coliform. In conclusion, the morphological state of UPEC has major impact on the host bladder response both when evaluating the number and the identity of altered host genes and pathways.

## Introduction

Urinary tract infection (UTI) is the second most common infectious disease in humans, following infections in the respiratory tract, and affects mainly women^1^. One reason for the high frequency of UTIs is that a large proportion (approximately 25–35%) of women treated for UTI will subsequently be afflicted by a recurrent infection within 6 months after the initial infection^1,2^. Uropathogenic *Escherichia coli* (UPEC) are responsible for the vast majority of UTIs in uncompromised patients and recurrent UTI is often caused by the same clone as the first infection^3,4^. This suggests that UPEC have evolved strategies to persist in the urinary tract and evade antibiotic treatment and host response mechanisms.

It is now well established that UPEC strains are able to invade host bladder epithelial cells where they can replicate and form biofilm-like intracellular bacterial communities. Studies in mouse bladders have suggested that UPEC undergo a cyclic cascade in which intracellular UPEC replicate and subsequently escape from the colonized bladder cells to enter a new cycle of infection^5–7^. The subsequent resurgence of these intracellular reservoirs is considered a possible cause of recurrent or chronic UTI^8^. Intracellular UPEC undergo morphological alterations characterized by transformation from a rod shape to a coccoid morphology that may follow by an additional transition into a filamentous form^6,7,9^. The filamentous, elongated morphology dominates during escape from the host bladder cells^7^, but re-infection of host cells is believed to occur first after reverting of filaments to a rod-shaped morphology^10^. Filamentous *E. coli* and intracellular colonized bladder epithelial cells have been observed in urine samples from women and children, suggesting that the UPEC cyclic cascade is present also in humans^8,11,12^. UPEC filamentation has been demonstrated in response to several stimuli such as components of the host defense system^9^, urine flow^10^ and beta-lactam antibiotics^13–15^.

Beta-lactam antibiotics, in particular aminothiazolyl cephalosporins like ceftibuten, induce *E. coli* filamentation by inhibiting penicillin-binding protein-3 that catalyses septa formation^13^. Extended-spectrum β-lactamases (ESBL) are enzymes that are able to hydrolyse various types of newer β-lactam antibiotics, including the oxyimino-cephalosporins (also known as the 3rd and 4th generation cephalosporins) and the monobactams^16^. The prevalence of ESBL-producing *E. coli* has increased dramatically all over the world during the past decades, and a majority of the ESBL-producing bacteria are isolated from urine samples from patients with UTI^17^. When exposed to beta-lactam antibiotics ESBL-producing UPEC may both resist the treatment and, in addition, transform into a filamentous morphology that may increase the chances for UPEC to persist in the bladder. Following antibiotic with-drawl, the filamentous form is rapidly reverted back to its normal coliform^15^. Thus, the filamentous and reverted morphology of UPEC may appear in the urinary tract as a result of the cyclic cascade associated with intracellular invasion and evasion but also as a result of antibiotic treatment.

The virulence and host activating properties of the different morphologies are largely unknown and need to be defined to fully understand the pathogenic potential of UPEC during their morphological switch. Two recent global gene expression studies of UPEC transition through the different morphological states revealed alterations in many genes, with the highest number of altered genes in the filamentous state^15,18^. Overall, the data suggested that the filamentous and reverted morphologies were associated with enrichment of genes involved in metabolic processes, cell division, cell adhesion and iron acquisition^15,18^. It has been suggested that the filamentous morphology may provide several advantages for UPEC e.g., an increased adhesion capacity to host cells by more contact points^9,10^, including an increased capacity of the filaments to resist liquid shear forces^10^, and protection against phagocyte killing^5,9^. Furthermore, genes associated with LPS biosynthesis were up-regulated in filamentous UPEC, but down-regulated in reverted bacteria^15^, suggesting that activation of LPS-dependent pro-inflammatory responses may differ between the morphologies.

Co-infection studies with assessment of host bladder cell responses are required to properly address the pathogenic potential of the filamentous and reverted UPEC forms during their morphological switch. In the present study we evaluate changes in gene expression in human primary bladder epithelial cells infected with normal coliform, filamentous and reverted UPEC.

## Material and methods

### Cell and bacterial culture

Human bladder epithelium progenitor cell line (HBEP.05, CELLnTEC Advanced Cell Systems AG, Bern, Switzerland) from a single donor was cultured in CnT-58 cell culture medium (CELLnTEC) supplemented with 100 U/mL penicillin and 100 μL/mg streptomycin (PEST) (Invitrogen Ltd, Paisley, UK) in a humidified atmosphere with 5% CO_2_ at 37 °C. At confluency the culture was differentiated into umbrella cell-like cells during four days using CnT-21 differentiation medium (CELLnTEC) supplemented with 1 mM CaCl2 in 6-well plates. The ESBL-producing *E. coli* (designated ESBL019) was obtained from the bacterial culture collection at Örebro University hospital, Sweden. Antimicrobial susceptibility testing was performed as recommended by the Swedish reference Group for Antibiotics (www.srga.org). This isolate has previously been characterized and determined to belong to the ST131 clone and to be multidrug resistant (CTX-M-15), including resistance to ceftibuten^19^. Bacteria were maintained on tryptic soy agar (Becton, Dickinson and Company, Sparks, MD). Prior to experiments, bacteria were inoculated in Difco Luria–Bertani (LB) broth (Lennox; Franklin Lakes, NJ, USA) and incubated at 37 °C aerobically on a shaker overnight. Bacteria were suspended in sterile phosphate buffered saline (PBS) prior to inoculation of cell culture medium (CCM) with or without ceftibuten (480 ng/mL).

### Cell stimulation

**Figure 1:**
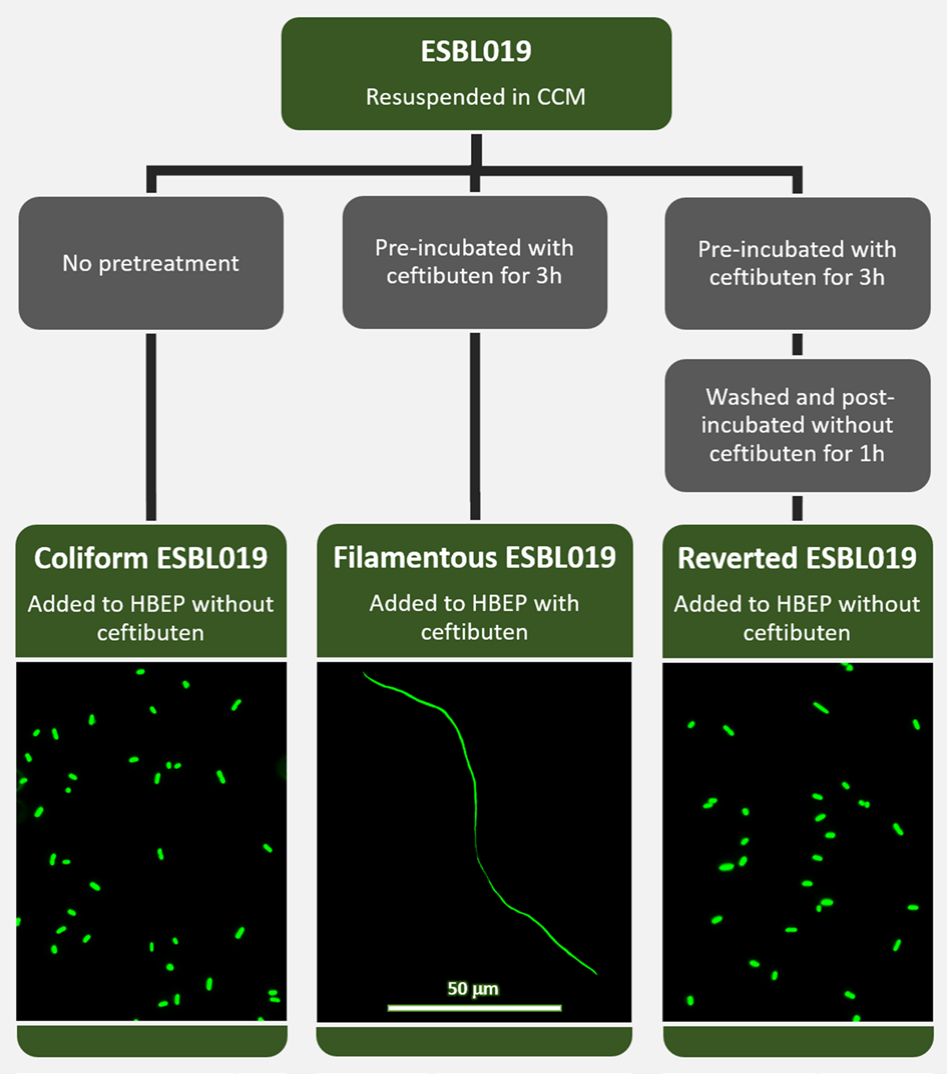
Summary of the experimental protocol and a visual demonstration of the three different morphological states of ESBL019. ESBL019 Coliform was grown for 3 h in cell culture medium (CCM) without ceftibuten, ESBL019 Filamentous was grown for 3 h in the presence of ceftibuten and ESBL019 Reverted was grown in the presence of ceftibuten for 3 h and then without ceftibuten for 1 h to allow its reversion. The bacteria were thereafter added to HBEP cells, in 6-well plates, in the presence or absence of ceftibuten as indicated. The lower panel demonstrates the different morphological states of ESBL019 carrying an eGFP-plasmid (enhanced green fluorescence protein, kindly provided by Professor Philip Poole at University of Oxford, UK). Scale bar: 50 µm.

Three different morphologies of ESBL019 were prepared for infecting HBEP cells as previously described^15^. Briefly, ESBL019 Coliform was grown for 3 h in cell culture medium (CCM) without ceftibuten, ESBL019 Filamentous was grown for 3 h in the presence of ceftibuten (480 ng/ml) and ESBL019 Reverted was grown in the presence of ceftibuten (480 ng/ml) for 3 h and then without ceftibuten for 1 h to allow its reversion. The bacteria were thereafter added to HBEP cells, in 6-well plates, in the presence (ESBL019 Filamentous) or absence (ESBL019 Coliform, ESBL019 Reverted) of ceftibuten (480 ng/ml). The protocol and the reversible cell morphology transition are shown in Fig. 1.

Prior to inoculation, the bacterial concentrations of all the morphologies were adjusted in order to infect HBEP cells with MOI 10. Infected HBEP cells were incubated for 4 h in 6-well plates in a humified atmosphere with 5% CO_2_ at 37 °C after which supernatants and RNA were collected. Alterations in HBEP gene expression in response to the different morphological states of ESBL019 were evaluated in comparison to unstimulated control cells incubated in CCM without ceftibuten. The bacteria from these experiments were harvested and used for transcriptional analysis as reported in a previous study^15^.

### RNA preparation and microarray

Total RNA from infected and unstimulated HBEP cells was isolated with RNeasy Mini Kit (Qiagen Technologies, Hilden, Germany) according to manufacturer instructions. RNA concentration and purity were measured using a Nano-Drop ND-1000 Spectrophotometer (Nano-Drop Technology Inc., Wilmington, DE, USA). All samples had OD260/280 and OD260/230 ratios above 1.9. RNA quality was further assessed using the Agilent RNA 6000 Nano Kit (Agilent) with 2100 Bioanalyzer (Agilent Technologies, Palo Alto, CA, USA) according to the manufacturer’s guidelines. RNA integrity number (RIN) values were > 9.0 for all samples. High-quality total RNA was used to prepare amplified Cy3-labeled cRNA with the one-color Low Input Quick Amp Labeling Kit (Agilent) according to manufacturer instructions. The cRNA concentration and labelling was determined by NanoDrop. Samples were hybridized to SurePrint G3 Human Gene Expression 8 × 60 k v2 Microarrays (Agilent) during 17 h at 65 °C in a Hybridization Oven (Agilent). The microarrays were washed and scanned with a G2565CA Microarray Scanner (Agilent) and image analysis as well as data extraction was assessed with Feature Extraction Software v10.7.3.1 (Agilent).

### Host cell invasion assay

Intracellular invasion was assessed by stimulating the human bladder epithelial cell line HBLAK (CELLnTEC Advanced Cell Systems AG, Bern, Switzerland) with the different bacterial morphological states at MOI 10 for 2 h at 37 °C in 24-well plates. The plate was washed with PBS after stimulation and the culturing medium was replaced with DMEM complemented with 2% FBS and 100 µg/ml gentamicin for an additional 2 h. The cells were thereafter washed, lysed with 0.1% Triton-X 100 in PBS, plated on TSA plates and grown overnight at 37 °C followed by CFU counting. Bacterial host cell invasion was presented as CFU/well.

### Data processing and statistical analysis

Microarray data analysis was performed using GeneSpring GX version 11.5 (Agilent) after per chip and 75th percentile shift gene normalization of samples (n = 4). Differentially expressed genes were subjected to Gene Ontology enrichment analysis in GeneSpring and to functional analyses through Ingenuity Pathway Analysis (IPA, QIAGEN Inc., https://www.qiagenbioinformatics.com/products/ingenuitypathway-analysis) with Core analyses for enrichments in canonical pathway, downstream functions and upstream regulators. For analyzing differences between groups one-way analysis of variance (ANOVA) parametric test was used. Significant entities (q < 0.05) were obtained by Tukey HSD post hoc test followed by Bonferroni multiple testing correction and a fold change cut-off ≥ 2. Significant GO term enrichment was set at a *p*-value < 0.05.

## Results

### Gene expression alterations in response to different morphological states of ESBL019

**Figure 2:**
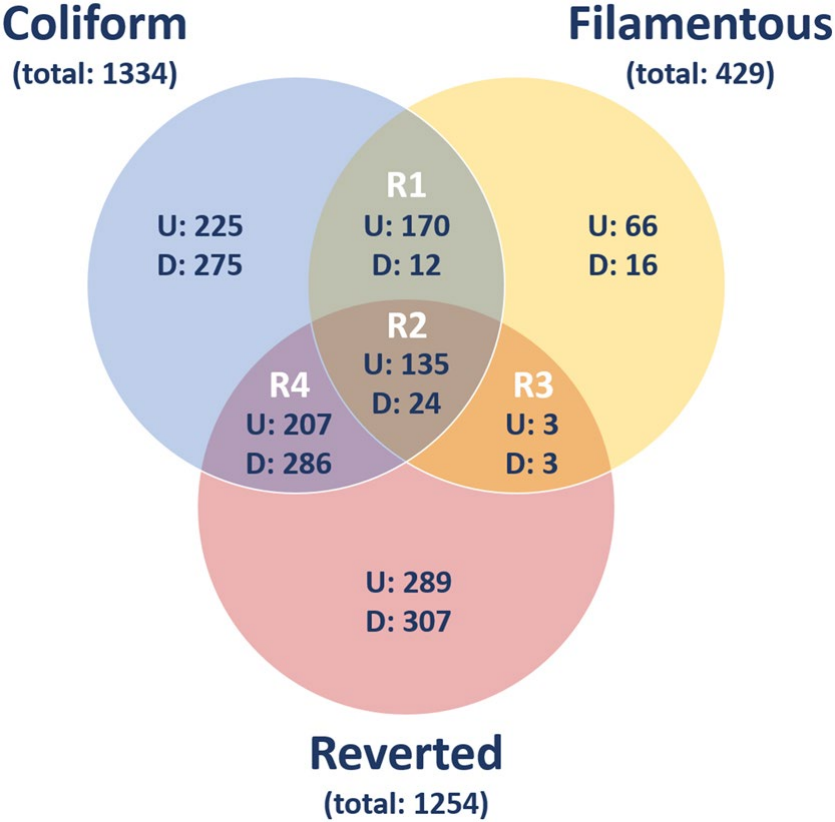
Venn diagram of differentially expresses gene entities in HBEP cells stimulated by different morphological states of ESBL019 compared to unstimulated controls. Shown in blue; HBEP cells stimulated by the Coliform, in yellow; HBEP cells stimulated by Filamentous bacteria and pink; HBEP cells stimulated by Reverted bacteria. Up and down regulated entities are designated U and D respectively (n = 4 in each group). Overlapping regions represent entities that are present in Coliform and Filamentous (R1 + R2), Filamentous and Reverted (R2 + R3) or Coliform and Reverted (R2 + R4).

Microarray analysis was performed on mRNA isolated from HBEP cells exposed to bacteria following their morphological transition from coli form to a filamentous form and when reverted to their original coli form following antibiotic withdrawal. In total 2018 entities were differentially expressed (*p* < 0.05) with at least a ≥ twofold change compared to unstimulated control cells. All three morphological states of the bacteria, Coliform, Filamentous and Reverted, induced definite changes in gene expressions in HBEP cells with 737, 374 and 634 up regulated and 597, 55 and 620 down-regulated gene expressions, respectively (Table 1). Differentially expressed gene entities within each group are visualized using a Venn diagram (Fig. 2). When comparing unstimulated HBEP cells incubated with or without ceftibuten only a few, in comparison to the number of differences seen with bacterial exposure, were seen. In total 72 entities were differentially expressed and of these 50 were entities annotated to uncharacterized locus areas and hypothetical proteins. The remaining 22 entities were in general transcript variants annotated to genes with a low fold change and not included among genes found in relation to bacterial infection.

**Table 1:** Differentially expressed gene entities with a ≥  2 fold change.

	Coliform	Shared gene entities (R1 + R2)	Filamentous	Shared gene entities (R2 + R3)	Reverted	Shared gene entities (R2 + R4)
Up-regulated	737	305	374	138	634	342
Down-regulated	597	36	55	27	620	310
Total	1334	341	429	165	1254	652

Taken together 341 altered gene entities are shared between Coliform and Filamentous (R1 + R2) with 305 up-regulated and 36 down-regulated entities, and 165 gene entities were shared between Filamentous and Reverted (R2 + R3) with 138 up-regulated and 27 down-regulated entities. In addition, 652 gene entities were shared between Coliform and Filamentous (R2 + R4) with 342 up-regulated and 310 down-regulated entities (Table 1, Fig. 2). A total of 159 gene entities were shared between all three groups with 135 up- regulated and 24 down-regulated entities (Fig. 2). Here, we identified a set of strongly up-regulated genes with a fold change > 100, including IL17C, TNFAIP6, TNF, IL20, CXCL2, CXCL3, IL6, CXCL8, ICAM1, CXCL1, IL23A, CSF3, CCL20, CSF2, PTX3 (Table 2). Up-regulation of these genes, except for ICAM1, was markedly higher in HBEP cells stimulated with the Coliform compared to cells stimulated with Filamentous and Reverted bacteria (Table 2). For a summary of the altered unique and shared gene entities see Supplement (Tables S1–S6).

**Table 2:** A comparative analysis of shared gene entities altered in HBEP cells after stimulation with all three morphologies (Coliform, Filamentous, Reverted).

Gene symbol	Coliform vs C	Filamentous vs C	Reverted vs C	Description
IL17C	2406	1371	11.5	Homo sapiens interleukin 17C
TNFAIP6	1231	398	7.3	Homo sapiens tumor necrosis factor alpha-induced protein 6
TNF	1162	49.7	68.8	Homo sapiens tumor necrosis factor
IL20	442	75.4	19.5	Homo sapiens interleukin 20
CXCL2	393	22.1	61.2	Homo sapiens chemokine (C-X-C motif) ligand 2
CXCL3	339	13.6	19.9	Homo sapiens chemokine (C-X-C motif) ligand 3
IL6	275	23.8	16.1	Homo sapiens interleukin 6
CXCL8	274	49.0	24.7	Homo sapiens chemokine (C-X-C motif) ligand 8
ICAM1	252	334	3.9	Homo sapiens intercellular adhesion molecule 1
CXCL1	222	66.3	13.4	Homo sapiens chemokine (C-X-C motif) ligand 1
ZNF485	− 3.8	− 2.2	− 2.8	Homo sapiens zinc finger protein 485
DFFB	− 4.1	− 2.0	− 2.8	Homo sapiens DNA fragmentation factor, 40 kDa, beta polypeptide (caspase-activated DNase), transcript variant 1
ID3	− 4.6	− 3.2	− 2.5	Homo sapiens inhibitor of DNA binding 3, dominant negative helix-loop-helix protein
ZNF214	− 4.6	− 2.7	− 3.2	Homo sapiens zinc finger protein 214
ZNF239	− 4.7	− 2.6	− 2.2	Homo sapiens zinc finger protein 239, transcript variant 1
IRX5	− 5.1	− 3.2	− 2.1	Homo sapiens iroquois homeobox 5, transcript variant 1
CBX2	− 5.3	− 2.5	− 5.6	Homo sapiens chromobox homolog 2, transcript variant 1
RAB3A	− 6.9	− 2.6	− 4.2	Homo sapiens RAB3A, member RAS oncogene family
SOX2	− 9.2	− 3.3	− 3.0	Homo sapiens SRY (sex determining region Y)-box 2
ZFP37	− 14.3	− 3.1	− 5.0	Homo sapiens ZFP37 zinc finger protein, transcript variant 3

Data are expressed as fold change compared to unstimulated control cells (C). The top 10 up-regulated and down-regulated genes are shown.

### Gene ontology analysis

Gene ontology analysis was performed on differentially expressed entities after stimulation with bacteria from each of the three morphological states. In total, 256 (Coliform), 387 (Filamentous) and 286 (Reverted) significantly (*p* < 0.05) enriched gene ontologies were identified. Of these, 18 (Coliform), 67 (Filamentous) and 27 (Reverted) gene ontologies were uniquely enriched (Tables S7–S9). Two gene ontologies (positive regulation of leukocyte chemotaxis and negative regulation of cell proliferation) were enriched in all three morphological states (Table S13). The number of enriched gene ontologies shared between two morphological states was 6 (Coliform + Filamentous), 9 (Coliform + Reverted) and 5 (Filamentous + Reverted) (Tables S10–S12).

### Upstream regulators

**Figure 3:**
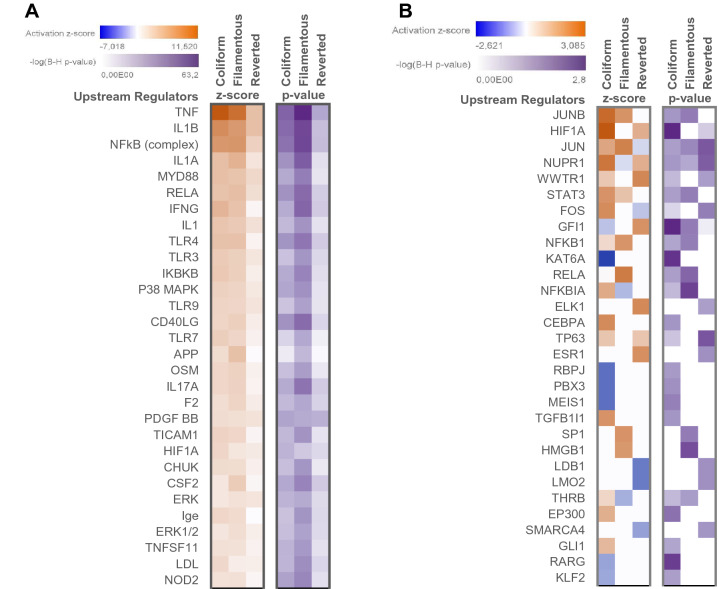
A comparative analysis of (**A**) shared upstream regulators altered in HBEP cells after stimulation with all three morphologies (Coliform, Filamentous, Reverted) compared to unstimulated control cells, (**B**) upstream regulators for gene entities that were uniquely altered in HBEP cells after stimulation with only one of the three morphologies (only Coliform, only Filamentous, only Reverted) compared to unstimulated control cells. The top 30 upstream regulators are shown, and data are presented as activation z-score and − log B-H corrected *p*-value.

**Figure 4:**
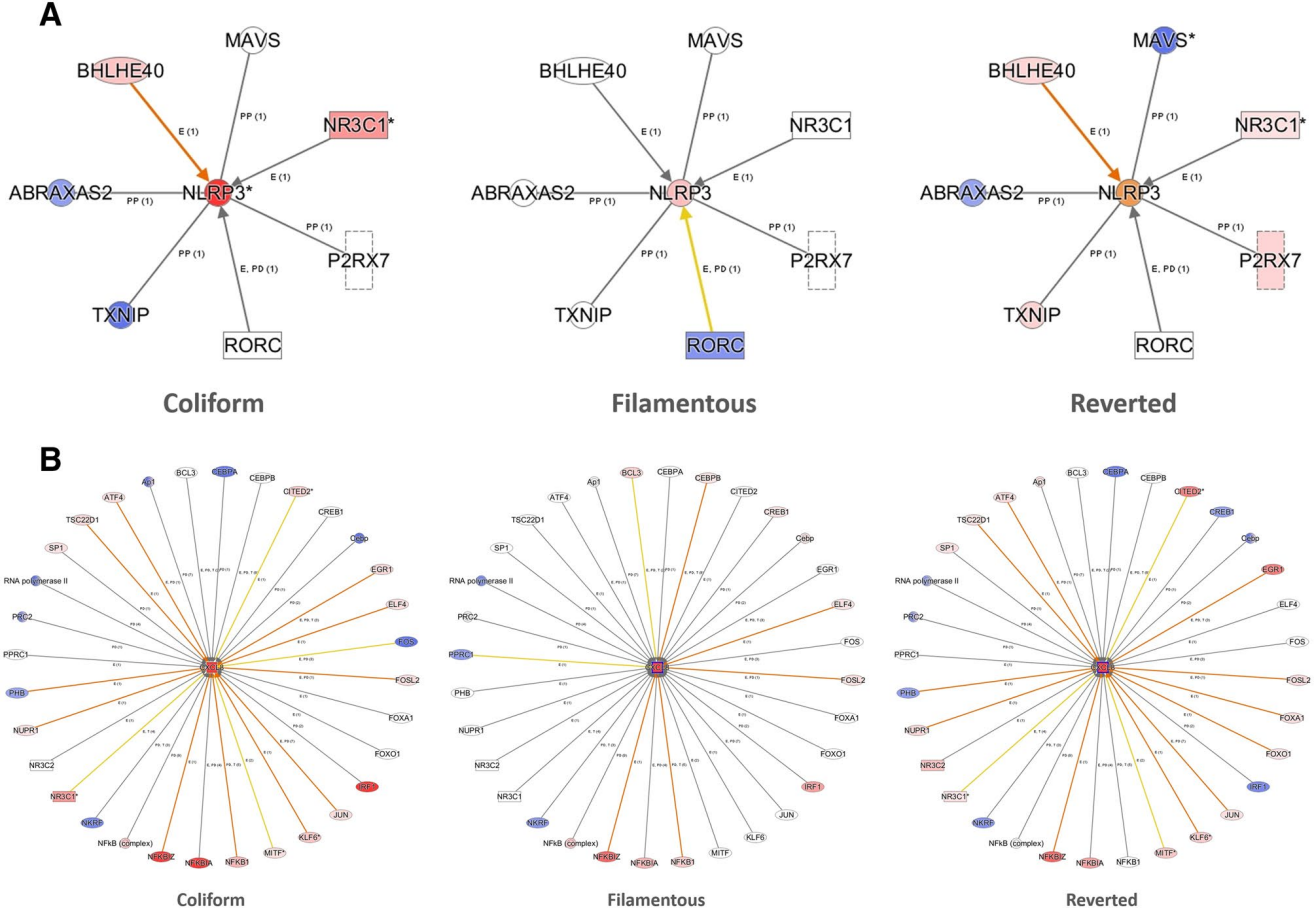
Pathway analysis showing upstream regulators associated with gene expression of (**A**) NLRP3 and (**B**) CXCL8. Red regulators are upregulated compared to unstimulated control cells while blue are downregulated. Red/orange lines indicate that the regulator leads to a predicted activation of gene expression while yellow lines indicate disagreement between the state of the upstream regulator and the predicted function. Grey lines indicate that no activation pattern can be predicted.

Analysis of upstream regulators was applied to characterize the cascade of regulators that may affect gene expression. The top-scored activated upstream regulators are presented in Fig. 3A. TNF, IL1B and IL1A are differently activated genes that are also identified here as key upstream regulators of gene expression. The predicted activation score of the top-scored upstream regulators was in general lower for cells stimulated with Reverted bacteria than for the other two morphologies (Fig. 3A). Analysis of upstream regulators for gene entities that were uniquely altered i.e., in response to only one of the morphological states are shown in Fig. 3B. These data demonstrate that the activation score pattern for some regulators is comparable but that many of the regulators show a specific activation score pattern for the group of uniquely altered gene entities associated with the respective morphological states. The upstream regulators that are predicted to regulate gene expression of NLRP3 (only significantly increased in HBEP cells stimulated with the Coliform) and CXCL8 (significantly increased in all three morphological states) are shown in Fig. 4. A general observation for these genes is that the number of significantly altered upstream regulators, as well as their predicted activation of the respective genes, are lower in HBEP stimulated with the Filamentous form.

### Pathway analysis

A canonical pathway analysis was performed for enrichment of altered gene entities in functional pathways with a cut-off of at least two matched entities in each pathway. Curation for exclusion of redundant pathways was made.

**Figure 5:**
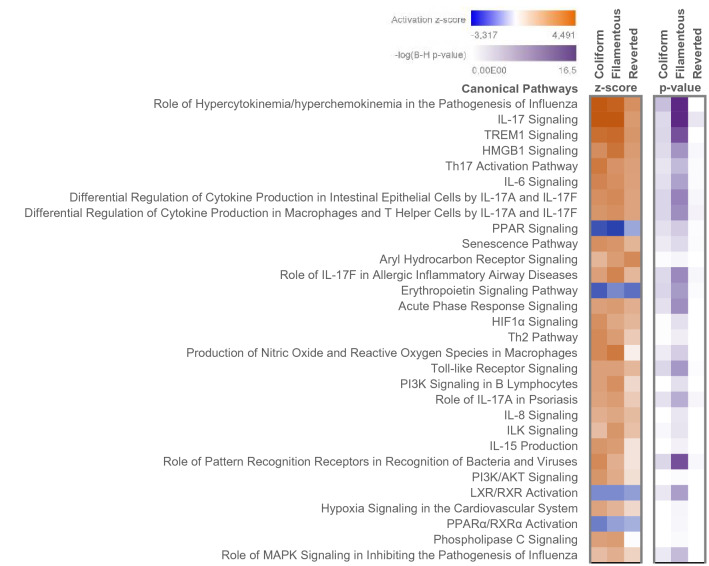
A comparative analysis of canonical pathways showing enrichment of altered gene entities in functional pathways in HBEP cells stimulated with Coliform, Filamentous and Reverted bacteria compared to unstimulated control cells. The top 30 activated pathways are shown, and data are presented as activation z-score and − log B-H corrected *p*-value.

The number of significantly changed canonical pathways was higher in HBEP cells stimulated with Filamentous bacteria (138 pathways), than in cells stimulated with the Coliform (83 pathways) or Reverted form (32 pathways). The top-scored significantly changed canonical pathways in HBEP cells stimulated with the different morphologies are shown in Fig. 5. Most of the pathways was activated (positive z-score), although PPAR and erythropoietin signalling pathway showed decreased activity in cells stimulated with all three morphologies (Fig. 5). Noteworthy, pathways related to IL-17/IL-17A/IL-17F-signalling, TREM1 and HMBG1 signalling were found among the top-scored pathways in all of the morphological states.

### Invasion of host bladder cells

**Figure 6:**
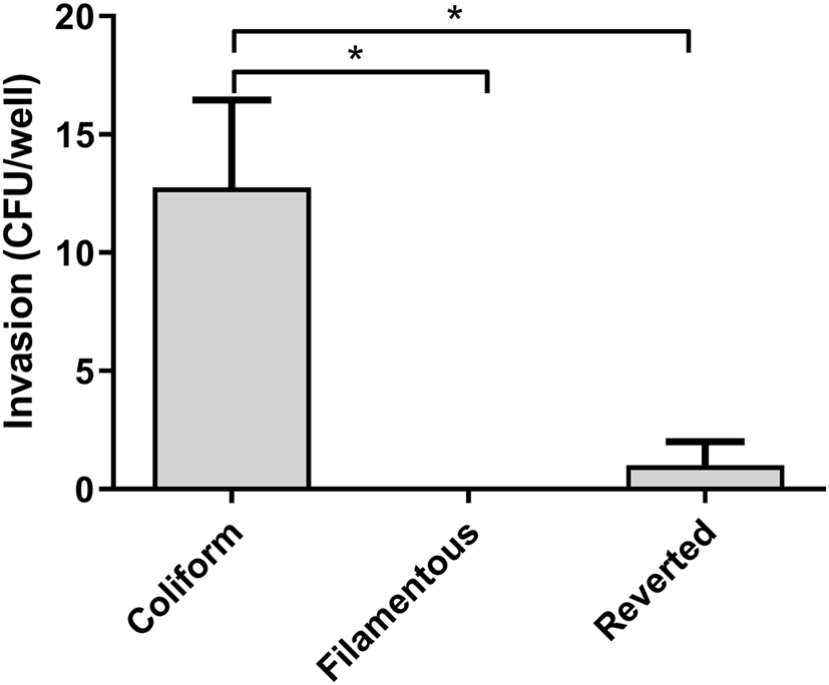
The human bladder epithelial cell line HBLAK was infected in 24-well plates with Coliform, Filamentous and Reverted bacteria followed by evaluation of bacterial invasion. Bacterial invasion was assessed and quantified as the number of intracellular bacteria (CFU)/well. Data are presented as mean ± SEM of four independent experiments. Statistical significance *p < 0.05.

The ability of the different bacterial morphologies to invade bladder epithelial cells was assessed by quantifying the number of intracellular bacteria after lysis of cells. Intracellular bacteria were detected in all experiments using Coliformed bacteria (13 ± 3.7 CFU/well). However, filamentous bacteria were unable to invade bladder cells, and the number of intracellular bacteria was markedly lower in cells infected with the Reverted form compared to the Coliform (Fig. 6).

## Discussion

UPEC pathogenesis involves a cyclic transition between extra- and intracellular localisation, along with a synchronized transition to filaments that subsequently reverts back to normal rod-shaped bacteria with a potential to re-infect host bladder cells^5,7^. In addition, and as applied in the present study, an extensive formation of bacterial filaments can also occur as a result of sub-lethal concentrations of antibiotics^13–15^. Previous studies have mainly focused on characteristics of the different bacterial morphologies^15,18^, while the host cell response following infection with the different morphologies have not been determined. In the present study we used human primary bladder epithelial cells (HBEP), instead of more commonly used bladder cancer epithelial cell lines, in order to increase the biological relevance of the host-bacteria interactions. However, a limitation using this one donor cell line is that the data is based on the cell responses from one donor, which may be different from the cell response obtained from different donors. The number of significantly induced changes in gene expressions in HBEP cells following infection with Coliform and Reverted bacteria were comparable, while the number of changed gene entities following stimulation with Filamentous bacteria were lower. An evaluation of overlapping gene entities showed that HBEP cells stimulated with Coliform and Reverted bacteria had the highest number of shared gene entities, while cells stimulated with Filamentous and Reverted bacteria had few uniquely shared gene entities. Taken together, this suggests that Filamentous bacteria are least prone to stimulate transcriptional activity of host bladder epithelial cells (reflected in a lower number of significantly altered genes) and that the number of altered genes in response to the normal Coliform and the Reverted form are more comparable. Our previous study focusing on UPEC global gene expression and virulence-associated genes during morphological transition, under the same antibiotic-induced conditions as the present study, showed that Filamentous UPEC underwent a more pronounced alterations in transcriptional activity than the Reverted form^15^. Merged with the results from the present study it seems that the profound transcriptional alterations of Filamentous UPEC^15^ result in a setback of host-activating properties. Importantly, our previous study on global gene expression of Filamentous and Reverted UPEC was performed in the presence of HBEP cells. This enables us to directly transfer knowledge on virulence properties of the Filamentous and Reverted form from the previous study to the present study when elucidating differences in host-activating properties of morphological diverse UPEC.

Although the number of induced gene entities was lower in cells stimulated with Filamentous bacteria, the number of enriched gene ontology classes was higher in cells stimulated with Filamentous bacteria than in cells stimulated with Coliform and Reverted bacteria. This suggests that Filamentous bacteria have diverse effects and affect a variety of different classes of host genes. Overall, the majority of the identified changes in gene ontologies were associated with immune system processes, cell signalling and cytokine/chemokine production, which is an expected finding. Two gene ontologies, positive regulation of leukocyte chemotaxis and negative regulation of cell proliferation, were enriched in cells stimulated with all three morphological states. These gene ontologies include many genes that are associated with host immune responses^20,21^ such as CXCL1, CXCL2, CXCL3, CXCL8, IL-6, IL17C and TNF, demonstrating that all morphological states can evoke a host immune response of bladder epithelial cells. However, it was evident from analysis of the transcriptional activity of the individual genes that the degree of host activation markedly differed between the three morphological states. The normal Coliform was often superior in stimulating transcriptional activity in HBEP cells compared to the Filamentous and Reverted form. UPEC depend on fimbriae for adhesion and invasion of bladder epithelial cells^22^, but the decreased activation of host immune factors found in the present study is unlikely to be explained by decreased adhesion of Filamentous and Reverted bacteria to HBEP cells. Filamentous and/or Reverted UPEC appear to maintain their functional adhesion properties^9,10,15^, and up-regulate P- and type-1 fimbriae-associated genes^15^, suggesting that the morphological switch do not attenuate the ability of the bacteria to adhere to host cells. Nevertheless, although the Filamentous and Reverted UPEC can adhere there are other factors of importance for host activation that may be depressed. We found in the present study that the Filamentous form was unable to invade host bladder cells. This emphasizes that the filamentous phase of UPEC infection needs to be transient and reversible given that a secondary host cell re-infection is believed to occur first after reverting of filaments to a rod-shaped morphology^10^. In our experimental set-up, the Reverted form was evaluated 1 h after antibiotic removal when the bacteria, based on microscopical inspection, appeared to have retained their normal rod-shaped form. However, it is obvious that the Reverted form in our study is rather in a transitory state than fully reverted given its compromised host-activating properties, including a low host invasion capacity. Thus, the overall lower transcriptional activation evoked by Filamentous and Reverted UPEC may be related to the impaired host cell invasion capacity of these morphological forms.

The endotoxin LPS mediates multiple aspects of UPEC pathogenesis by interacting with the Toll-like receptor 4 (TLR4), which subsequently activates the innate immune response and release proinflammatory cytokines^23^. Our previous study demonstrated that genes associated with LPS biosynthesis were up-regulated in the Filamentous bacteria, but down-regulated in Reverted bacteria^15^. In the present study, analysis of upstream regulators revealed a lower predicted activation z-score for TLR4 in HBEP cells stimulated with Reverted bacteria, while the activation score was comparable in cells stimulated with Coliform and Filamentous bacteria. HBEP is a low LPS-responsive cell line (unpublished observations) and, in combination with a variable expression of LPS and TLR4 among the morphological states, it is not likely that the depressed host activation by Filamentous and Reverted bacteria are explained by alterations in TLR4 signalling. Interestingly, the adhesion molecule ICAM-1 showed a higher activity in cells stimulated by Filamentous than Coliform bacteria. UPEC enhance the expression of ICAM-1 on urinary tract epithelial cells to facilitate neutrophil migration^24^, and a more pronounced physical interaction between the elongated filaments and host cells^9,10,15^ may possibly trigger an increased transcription of ICAM-1 by HBEP cells.

The cell division gene *damX*^18^ and SulA, an effector molecule in the bacterial SOS response^9^, have been implicated as a key inducer of UPEC filamentation. Deletion of *damX* or *sulA* abrogated both UPEC filamentation and the ability to establish murine urinary tract infection^9,18^, suggesting that the ability to transiently switch into alternative morphologies may be an advantage for uropathogenesis. Our previous study has shown that ceftibuten-induced UPEC filamentation is associated with increased expression of *sulA*^15^, demonstrating that the filaments formed by ceftibuten in vitro show similarities with the morphological alterations that occur in vivo*.* A weak activation of the host immune system, as noted for Filamentous and Reverted bacteria, may be considered as an advantage for the bacteria since migration of phagocytic cells, like neutrophils, depend on an activated host response and secreted chemokines^20,21^. UPEC have evolved different mechanisms to suppress host recognition and activation e.g., by secreting TcpC that binds to MyD88 and inhibits down-stream signalling and cytokine responses by host cells in the urinary tract^25^. However, the expression of *tcpC* was found to be significantly downregulated in the Filamentous and Reverted forms compared to the normal Coliform^15^, which argues against an involvement of TcpC. Nevertheless, it cannot be excluded that the weak stimulatory effect on cytokine gene expression demonstrated in the present study by the Filamentous and Reverted forms involves an active suppression of host activation. Pro-inflammatory soluble cytokines that are generated by immune system cells in response to bacterial infection may also, through autocrine and paracrine mechanisms, act as upstream regulators of gene expression^26^. Our data showed that the cytokines TNF, IL1B and IL1A, which were all differently activated gene entities, were highly ranked among the upstream regulators. Thus, the overall lower primary activation of many HBEP genes in response to Filamentous and Reverted bacteria may, therefore, also affect the capacity of these cytokines to act as upstream regulators of other target molecules. Taken together, the results suggest that the Filamentous and Reverted morphological states evoke an overall weaker host immune response than the normal Coliform, but the underlying mechanisms needs to be further elucidated.

Some of the top-scored canonical pathways such as IL-6, TLR and IL-8 signalling are all pathways with an established connection to mechanisms underlying UTI pathogenesis^20,21^. Interestingly, several pathways related to IL-17-signalling were among the top-scored pathways in all three morphological states. The importance of IL-17A for innate immunity in UTI is demonstrated by a defect in acute clearance of UPEC and higher bacterial burden in IL-17A-deficient mice^27,28^. Also, the known action of IL-17 in cell recruitment and immune regulation^29^ imply that this cytokine is required to fine tune innate cellular defenses to UPEC in the bladder. IL-17C was the most upregulated gene in cells stimulated with Coliform and Filamentous bacteria, while a considerably lower expression was noted in response to Reverted bacteria. This is supported by data from the canonical pathway analysis demonstrating a considerable higher activation of ¨IL-17 Signaling¨ in Coliform and Filamentous bacteria than in the Reverted form. The danger-associated molecules high-mobility group box 1 (HMGB1) signalling and triggering receptor expressed on myeloid cells 1 (TREM1) signalling were other canonical pathways that displayed high activation scores. HMGB1 has many functions in the cell, including a proinflammatory role when released into the extracellular environment by passive release from necrotic cells or in an active manner following cell stimulation with e.g., LPS, IL-1β and TNF-α^30^. In the urinary tract, HMGB1 has been mainly been associated with the progression of urothelial carcinoma^31^, but bacterial infection of the urinary tract also stimulates increased expression of HMGB1^32^. Besides its own direct intracellular signaling pathway, TREM1 activation cross-talks with intracellular signalling pathways of several TLRs and can thereby contribute to and amplify the magnitude of inflammation^33^. A soluble form of TREM1 is shed from host cells and several studies have investigated the potential use of TREM-1 as a diagnostic marker for UTI^34,35^. Taken together, the pathway analysis demonstrated that the number of enriched pathways, and often the activation scores, were lowest in HBEP cells stimulated by the Reverted form. The Reverted form has undergone intense morphological transitions which appear to result in diminishing host activating properties. Both the number of significantly changed canonical pathways and gene ontology classes was highest in HBEP cells stimulated with Filamentous bacteria. Hence, the filamentous form may due to its exaggerated size be attributed to enhanced host recognition and surveillance. Moreover, our previous findings have shown that the filamentation process per se is associated with increased release of endotoxins and ATP into the cell supernatant, and that these soluble bacterial products are able to stimulate cytokine secretion from bladder epithelial cells^14^. Thus, the widespread activating response caused by the filamentous form may involve enhanced release of various extracellular host activating factors during the morphological transition into the filamentous form.

In conclusion, we have shown that the host bladder response to infection with different morphological states of UPEC, proposed to replicate the uropathogenic cascade, exhibit differences both in the number and identity of altered host genes and pathways. Re-infection of host bladder cells, as a potential cause of recurrent UTI, is likely to be a complex process and the outcome controlled both by the transient morphological state of the bacteria and the host immune response.

## Supplementary Information

Supplementary Information 1.

Supplementary Tables.

## Data Availability

All data generated or analysed during this study are included in this published article (and its Supplementary Information files).
