# Supplementary material for: Transcriptional alterations in bladder epithelial cells in response to infection with different morphological states of uropathogenic Escherichia coli
Source: Sci Rep. 2022 Jan 11;12:486. doi: 10.1038/s41598-021-04396-0 (PMC8752619; doi:10.1038/s41598-021-04396-0)
Supplement: Supplementary file 1 — Supplementary Information 1. [file 41598_2021_4396_MOESM1_ESM.docx]

**Table S1**: Altered gene entities in HBEP cells present only after stimulation with Coliform bacteria. Data are expressed as fold change compared to unstimulated control cells (C). Top 10 upregulated and downregulated genes are shown.

| **Gene symbol** | **Coliform vs C** | **Description** |
| --- | --- | --- |
| **RNA5-8C5** | 31.0 | Homo sapiens RNA, 5,8S ribosomal 5 |
| **CCL3** | 23.8 | Homo sapiens chemokine (C-C motif) ligand 3 |
| **TAGAP** | 21.4 | Homo sapiens T-cell activation RhoGTPase activating protein, transcript variant 2 |
| **NLRP3** | 11.0 | Homo sapiens NLR family, pyrin domain containing 3, transcript variant 6 |
| **NFKBID** | 7.1 | Homo sapiens nuclear factor of kappa light polypeptide gene enhancer in B-cells inhibitor |
| **ZCCHC6** | 6.8 | zinc finger, CCHC domain containing 6 |
| **OSMR** | 6.5 | Homo sapiens oncostatin M receptor, transcript variant 2 |
| **NPPC** | 6.5 | Homo sapiens natriuretic peptide C |
| **SNORD42A** | 6.5 | Homo sapiens small nucleolar RNA, C/D box 42A |
| **ID2** | 6.4 | Homo sapiens inhibitor of DNA binding 2, dominant negative helix-loop-helix protein |
| **ZKSCAN7** | -3.6 | Homo sapiens zinc finger with KRAB and SCAN domains 7, transcript variant 1 |
| **ARL4D** | -3.8 | Homo sapiens ADP-ribosylation factor-like 4D |
| **GXYLT1P3** | -3.9 | Homo sapiens glucoside xylosyltransferase 1 pseudogene 3 |
| **ZNF441** | -3.9 | Homo sapiens zinc finger protein 441 |
| **FRAT1** | -4.1 | Homo sapiens frequently rearranged in advanced T-cell lymphomas 1 |
| **ZNF30** | -4.2 | Homo sapiens zinc finger protein 30 (ZNF30), transcript variant 3 |
| **ARL4A** | -4.5 | Homo sapiens ADP-ribosylation factor-like 4A, transcript variant 1 |
| **ZNF780B** | -4.6 | Homo sapiens zinc finger protein 780B |
| **SOX7** | -4.9 | Homo sapiens SRY (sex determining region Y)-box 7 |
| **MYC** | -5.6 | Homo sapiens v-myc avian myelocytomatosis viral oncogene homolog |

**Table S2:** Altered gene entities in HBEP cells present only after stimulation with Filamentous bacteria. Data are expressed as fold change compared to unstimulated control cells (C). Top 10 upregulated and downregulated genes are shown.

| **Gene symbol** | **Filamentous vs C** | **Description** |
| --- | --- | --- |
| **IL19** | 90.6 | Homo sapiens interleukin 19, transcript variant 1 |
| **S100A7A** | 12.7 | Homo sapiens S100 calcium binding protein A7A |
| **ADAMTS4** | 6.1 | Homo sapiens ADAM metallopeptidase with thrombospondin type 1 motif, 4 |
| **CCL2** | 5.4 | Homo sapiens chemokine (C-C motif) ligand 2 |
| **TMPRSS2** | 4.9 | Homo sapiens transmembrane protease, serine 2, transcript variant 2 |
| **NES** | 4.7 | Homo sapiens nestin |
| **PRSS22** | 4.6 | Homo sapiens protease, serine, 22 |
| **PPFIA4** | 4.5 | protein tyrosine phosphatase, receptor type, f polypeptide (PTPRF), interacting protein (liprin), alpha 4 |
| **SAA4** | 4.2 | Homo sapiens serum amyloid A4, constitutive |
| **NTN1** | 3.8 | Homo sapiens netrin 1 |
| **TFAP2E** | -2.0 | Homo sapiens transcription factor AP-2 epsilon (activating enhancer binding protein 2 epsilon) |
| **SGK223** | -2.0 | Homo sapiens homolog of rat pragma of Rnd2 |
| **PLD6** | -2.1 | Homo sapiens phospholipase D family, member 6 |
| **SPRY1** | -2.1 | Homo sapiens sprouty homolog 1, antagonist of FGF signaling (Drosophila), transcript variant 2 |
| **E2F5** | -2.1 | Homo sapiens E2F transcription factor 5, p130-binding, transcript variant 1 |
| **RGS16** | -2.1 | Homo sapiens regulator of G-protein signaling 16 |
| **SPERT** | -2.3 | Homo sapiens spermatid associated, transcript variant 1 |
| **GCSAM** | -2.9 | Homo sapiens germinal center-associated, signaling and motility, transcript variant 3 |
| **ANKRD31** | -2.9 | Homo sapiens ankyrin repeat domain 31 |
| **CDON** | -3.0 | Homo sapiens cell adhesion associated, oncogene regulated, transcript variant 2 |

**Table S3:** Altered gene entities in HBEP cells present only after stimulation with Reverted bacteria. Data are expressed as fold change compared to unstimulated control cells (C). Top 10 up-regulated and down-regulated genes are shown.

| **Gene symbol** | **Reverted vs C** | **Description** |
| --- | --- | --- |
| **ECT2L** | 9.1 | Homo sapiens epithelial cell transforming 2 like, transcript variant 1 |
| **SPATA13** | 9.0 | spermatogenesis associated 13 |
| **ZNF582** | 8.5 | Homo sapiens zinc finger protein 582 |
| **CTGF** | 8.0 | Homo sapiens connective tissue growth factor |
| **ZNF608** | 7.5 | Homo sapiens zinc finger protein 608 |
| **HIST2H2BF** | 7.3 | histone cluster 2, H2bf |
| **VAV3** | 6.9 | Homo sapiens vav 3 guanine nucleotide exchange factor, transcript variant 1 |
| **BMPER** | 6.1 | Homo sapiens BMP binding endothelial regulator |
| **LIMS3L** | 6.0 | Homo sapiens LIM and senescent cell antigen-like domains 3-like, transcript variant 2 |
| **WEE1** | 5.4 | Homo sapiens WEE1 G2 checkpoint kinase, transcript variant 1 |
| **SERPIND1** | -4.6 | Homo sapiens serpin peptidase inhibitor, clade D (heparin cofactor), member 1 |
| **TRIM25** | -5.0 | Homo sapiens tripartite motif containing 25 |
| **SCARNA22** | -5.6 | Homo sapiens small Cajal body-specific RNA 22 |
| **VTRNA1-3** | -5.9 | Homo sapiens vault RNA 1-3 |
| **VTRNA1-2** | -5.9 | Homo sapiens vault RNA 1-2 |
| **HMGA2** | -6.0 | Homo sapiens high mobility group AT-hook 2, transcript variant 1 |
| **CAPN15** | -6.1 | calpain 15 |
| **SNORA12** | -7.4 | Homo sapiens small nucleolar RNA, H/ACA box 12 |
| **BMP6** | -10.3 | Homo sapiens bone morphogenetic protein 6 |
| **CEMIP** | -11.3 | Homo sapiens cell migration inducing protein, hyaluronan binding, transcript variant 3 |

**Table S4.** A comparative analysis of shared gene entities altered in HBEP cells after stimulation with Coliform and Filamentous bacteria. Data are expressed as fold change compared to unstimulated control cells (C). Top 10 upregulated and downregulated genes are shown.

| **Gene symbol** | **Coliform vs C** | **Filamentous vs C** | **Description** |
| --- | --- | --- | --- |
| **MIR146A** | 75.5 | 39.4 | microRNA 146a |
| **C2CD4A** | 44.8 | 5.6 | Homo sapiens C2 calcium-dependent domain containing 4A |
| **NEURL3** | 43.4 | 22.0 | Homo sapiens neuralized E3 ubiquitin protein ligase 3, transcript variant 2 |
| **CCL412** | 43.3 | 70.9 | Homo sapiens chemokine (C-C motif) ligand 4-like 2, transcript variant CCL4L2b2 |
| **TRAF1** | 40.4 | 17.3 | Homo sapiens TNF receptor-associated factor 1, transcript variant 1 |
| **CCL5** | 37.2 | 13.0 | Homo sapiens chemokine (C-C motif) ligand 5, transcript variant 1 |
| **C2CD4B** | 33.1 | 3.2 | Homo sapiens C2 calcium-dependent domain containing 4B |
| **PGLYRP2** | 30.9 | 16.5 | Homo sapiens peptidoglycan recognition protein 2 |
| **RND1** | 27.0 | 21.8 | Homo sapiens Rho family GTPase 1 |
| **MMP12** | 24.1 | 9.8 | Homo sapiens matrix metallopeptidase 12 (macrophage elastase) |
| **LOC344887** | -2.0 | -2.0 | Homo sapiens NmrA-like family domain containing 1 pseudogene |
| **LANCL2** | -2.0 | -2.5 | Homo sapiens LanC lantibiotic synthetase component C-like 2 (bacterial) |
| **PRICKLE1** | -2.2 | -2.8 | Homo sapiens prickle homolog 1 (Drosophila), transcript variant 1 |
| **CDK20** | -2.2 | -2.1 | Homo sapiens cyclin-dependent kinase 20, transcript variant 3 |
| **FANCF** | -2.4 | -2.1 | Homo sapiens Fanconi anemia, complementation group F |
| **IRX3** | -2.4 | -2.8 | Homo sapiens iroquois homeobox 3 |
| **JADE1** | -2.5 | -2.5 | Homo sapiens jade family PHD finger 1, transcript variant 2 |
| **DLX6** | -2.8 | -2.4 | Homo sapiens distal-less homeobox 6 |
| **B4GAT1** | -3.1 | -2.0 | Homo sapiens beta-1,4-glucuronyltransferase 1 |
| **HEXIM2** | -3.4 | -2.2 | hexamethylene bis-acetamide inducible 2 |

**Table S5.** A comparative analysis of shared gene entities altered in HBEP cells after stimulation with Coliform and Reverted bacteria. Data are expressed as fold change compared to unstimulated control cells (C). Top 10 upregulated and downregulated genes are shown.

| **Gene symbol** | **Coliform vs C** | **Reverted vs C** | **Description** |
| --- | --- | --- | --- |
| **AKR1C1** | 35.7 | 4.8 | aldo-keto reductase family 1, member C1 |
| **ZFP42** | 35.3 | 4.9 | ZFP42 zinc finger protein |
| **EDN2** | 31.1 | 20.5 | Homo sapiens endothelin 2, transcript variant 1 |
| **HILPDA** | 27.2 | 7.2 | Homo sapiens hypoxia inducible lipid droplet-associated, transcript variant 1 |
| **PTGS2** | 11.7 | 12.1 | Homo sapiens prostaglandin-endoperoxide synthase 2 (prostaglandin G/H synthase and cyclooxygenase) |
| **DLC1** | 11.6 | 2.2 | Homo sapiens DLC1 Rho GTPase activating protein, transcript variant 1 |
| **KRT37** | 8.0 | -2.0 | Homo sapiens keratin 37, type I |
| **NR4A2** | 7.7 | 15.1 | Homo sapiens nuclear receptor subfamily 4, group A, member 2 |
| **PHLDB2** | 7.6 | 23.1 | Homo sapiens pleckstrin homology-like domain, family B, member 2 |
| **SCNN1G** | 7.4 | 3.8 | Homo sapiens sodium channel, non voltage gated 1 gamma subunit |
| **VASN** | -7.2 | -5.2 | Homo sapiens vasorin |
| **ZFP82** | -7.3 | -5.6 | Homo sapiens ZFP82 zinc finger protein |
| **ZNF684** | -7.3 | -4.8 | Homo sapiens zinc finger protein 684 |
| **ID1** | -7.3 | -4.4 | Homo sapiens inhibitor of DNA binding 1, dominant negative helix-loop-helix protein, transcript variant 1 |
| **SNORD56B** | -7.4 | -4.6 | Homo sapiens small nucleolar RNA, C/D box 56B |
| **NFYA** | -7.9 | -6.7 | Homo sapiens nuclear transcription factor Y, alpha, transcript variant 1 |
| **ZNF709** | -10.6 | -8.1 | Homo sapiens zinc finger protein 709 |
| **NANOS1** | -13.8 | -17.7 | Homo sapiens nanos homolog 1 (Drosophila) |
| **HES5** | -17.9 | -5.5 | Homo sapiens hes family bHLH transcription factor 5 |
| **RASL11B** | -20.2 | -16.4 | Homo sapiens RAS-like, family 11, member B |

**Table S6.** A comparative analysis of shared gene entities altered in HBEP cells after stimulation with Filamentous and Reverted bacteria. Data are expressed as fold change compared to unstimulated control cells (C).

| **Gene symbol** | **Filamentous vs C** | **Reverted vs C** | **Description** |
| --- | --- | --- | --- |
| **SAMSN1** | 9.1 | -7.4 | Homo sapiens SAM domain, SH3 domain and nuclear localization signals 1, transcript variant 1 |
| **LMO2** | 5.1 | -2.3 | Homo sapiens LIM domain only 2 (rhombotin-like 1), transcript variant 1 |
| **PLAT** | 3.2 | -2.6 | Homo sapiens plasminogen activator, tissue, transcript variant 1 |
| **SOCS1** | 3.1 | -3.2 | Homo sapiens suppressor of cytokine signaling 1 |
| **CLDN3** | 2.8 | 3.8 | Homo sapiens claudin 3 |
| **FOSB** | 2.4 | 11.0 | Homo sapiens FBJ murine osteosarcoma viral oncogene homolog B, transcript variant 1 |
| **LYN** | 2.3 | -2.5 | Homo sapiens LYN proto-oncogene, Src family tyrosine kinase, transcript variant 1 |
| **OVOL1** | 2.0 | 3.0 | Homo sapiens ovo-like zinc finger 1 |
| **FIGN** | -2.1 | 2.5 | Homo sapiens fidgetin |
| **KRTAP4-1** | -2.2 | 2.3 | Homo sapiens keratin associated protein 4-1 |
| **C1QA** | -3.2 | -4.0 | Homo sapiens complement component 1, q subcomponent, A chain |
