# Supplementary material for: Transcriptional alterations in bladder epithelial cells in response to infection with different morphological states of uropathogenic Escherichia coli
Source: Sci Rep. 2022 Jan 11;12:486. doi: 10.1038/s41598-021-04396-0 (PMC8752619; doi:10.1038/s41598-021-04396-0)
Supplement: Supplementary file 2 — Supplementary Tables. [file 41598_2021_4396_MOESM2_ESM.docx]

**Table S7.** Enriched gene ontologies among significantly altered entities in HBEP cells stimulated by ESBL019 Coliform compared to unstimulated control cells. The top 10 most significantly enriched gene ontologies are shown.

| **Function** | **GO ID** | **GO Term** | **p-value** | **Counts in selection** | **Counts in total** | **Regulation up/down** |
| --- | --- | --- | --- | --- | --- | --- |
| Nucleic acid binding | 597 | RNA polymerase II regulatory region sequence-specific DNA binding | 0.004 | 41 | 396 | 32/8 |
| Immune system process | 31442 | granulocyte chemotaxis | 0.012 | 11 | 51 | 11/0 |
| Regulation of immune system process | 2021 | positive regulation of myeloid leukocyte differentiation | 0.004 | 11 | 45 | 6/5 |
| Regulation of cellular process | 43747 | regulation of leukocyte apoptotic process | 0.025 | 12 | 65 | 9/3 |
| Regulation of multicellular organismal process | 18988  1098 | positive regulation of cytokine biosynthetic process  negative regulation of cytokine production | 0.027  0.024 | 11  23 | 56  189 | 11/0  20/3 |
| Response to stimulus | 31278 | cellular response to hypoxia | 0.009 | 15 | 87 | 13/2 |
| Developmental  process | 29009 | morphogenesis of a branching epithelium | 0.020 | 21 | 162 | 8/13 |
| Cellular process | 24804  5667 | positive regulation of smooth muscle cell proliferation  cell death | 0.026  0.022 | 9  77 | 38  985 | 6/9  60/17 |

**Table S8.** Enriched gene ontologies among significantly altered entities in HBEP cells stimulated by ESBL019 Filamentous compared to unstimulated control cells. The top 10 most significantly enriched gene ontologies are shown.

| **Function** | **GO ID** | **GO Term** | **p-value** | **Counts in selection** | **Counts in total** | **Regulation up/down** |
| --- | --- | --- | --- | --- | --- | --- |
| Protein binding | 5511  3664 | chemokine activity  interleukin-1 receptor activity | 0.000  0.001 | 11  5 | 44  16 | 11/0  5/0 |
| Signaling | 29948  5178 | chemokine-mediated signaling pathway  I-kappaB kinase/NF-kappaB signaling | 0.000  0.000 | 9  8 | 44  50 | 9/0  8/0 |
| Response to stress | 4961  19531 | acute-phase response  defense response to bacterium | 0.000  0.002 | 8  13 | 39  178 | 8/0  13/0 |
| Regulation immune system process | 25926 | negative regulation of B cell activation | 0.000 | 7 | 30 | 7/0 |
| Regulation of metabolic process | 22329 | positive regulation of transcription by RNA polymerase II | 0.001 | 34 | 877 | 32/2 |
| Regulation of signaling | 38274 | regulation of NIK/NF-kappaB signaling | 0.001 | 8 | 54 | 8/0 |
| Regulation of multicellular organismal process | 14818 | positive regulation of chemokine production | 0.000 | 8 | 36 | 8/0 |

**Table S9.** Enriched gene ontologies among significantly altered entities in HBEP cells stimulated by ESBL019 Reverted compared to unstimulated control cells. The top 10 most significantly enriched gene ontologies are shown.

| **Function** | **GO ID** | **GO Term** | **p-value** | **Counts in selection** | **Counts in total** | **Regulation up/down** |
| --- | --- | --- | --- | --- | --- | --- |
| DNA binding | 598 | RNA polymerase II proximal promoter sequence-specific DNA binding | 0.000 | 34 | 273 | 31/3 |
| Transcription regulatory activity | 2653 | transcription corepressor activity | 0.001 | 28 | 211 | 27/1 |
| Lipid biosynthetic process | 862 | prostaglandin biosynthetic process | 0.011 | 7 | 21 | 3/4 |
| Cell transduction | 24264 | platelet-derived growth factor receptor signaling pathway | 0.004 | 9 | 31 | 8/1 |
| Response to external stimulus | 31047 | cellular response to lipopolysaccharide | 0.000 | 18 | 97 | 15/3 |
| Developmental  process | 17279 | tube development | 0.001 | 69 | 795 | 52/17 |
| Regulation of signalling | 17524  22673 | intracellular signal transduction  positive regulation of JNK cascade | 0.000  0.003 | 118  17 | 1547  103 | 70/48  12/5 |
| Regulation of multicellular organismal process | 12599 | regulation of ossification | 0.005 | 22 | 166 | 11/11 |
| Positive regulation of biological process | 1099 | positive regulation of cytokine production | 0.000 | 42 | 335 | 33/9 |

**Table S10.** Shared gene ontologies present among significantly altered entities in HBEP cells stimulated by the morphological states Coliform (C) and Filamentous (F) compared to unstimulated control cells.

| **Function** | **GO ID** | **GO Term** | **p-value** | **Counts in selection** | **Counts in total** |
| --- | --- | --- | --- | --- | --- |
| Protein binding | 21694 | CXCR chemokine receptor binding | 0.035  0.000 | C:6  F:8 | 17 |
| Immune system process | 21567 | innate immune response | 0.016  0.000 | C:66  F:43 | 800 |
| Signaling | 13811 | lipopolysaccharide-mediated signaling pathway | 0.000  0.000 | C:9  F:9 | 27 |
| Regulation of metabolic process | 21869 | positive regulation of nitric oxide biosynthetic process | 0.000  0.000 | C:9  F:7 | 31 |
| Regulation of response to stimulus | 25790 | positive regulation of inflammatory response | 0.001  0.001 | C:14  F:9 | 79 |
| Regulation of multicellular organismal process | 14851 | positive regulation of interleukin-6 production | 0.000  0.000 | C:10  F:8 | 49 |

**Table S11.** Shared gene ontologies present among significantly altered entities in HBEP cells stimulated by the morphological states Coliform (C) and Reverted (R) compared to unstimulated control cells.

| **Function** | **GO ID** | **GO Term** | **p-value** | **Counts in selection** | **Counts in total** |
| --- | --- | --- | --- | --- | --- |
| Cellular component | 3920 | nucleus | 0.000  0.000 | C:454  R:411 | 6484 |
| Transcription regulator activity | 813 | transcriptional activator activity, RNA polymerase II transcription regulatory region sequence-specific DNA binding | 0.038  0.041 | C: 27  R:26 | 248 |
|  | 674 | transcriptional repressor activity, RNA polymerase II proximal promoter sequence-specific DNA binding | 0.004  0.001 | C:16  R:17 | 91 |
| Protein binding | 3640 | cytokine activity | 0.000  0.000 | C:41  R:30 | 208 |
| Ion binding | 23155 | metal ion binding | 0.000  0.000 | C:302  R:251 | 3919 |
| Response to stimulus | 4962 | inflammatory response | 0.000  0.000 | C:63  R:47 | 414 |
| Signaling | 11008 | cytokine-mediated signalling pathway | 0.000  0.046 | C:46  R:35 | 380 |
| Regulation of cell death | 19826 | positive regulation of apoptotic process | 0.001  0.000 | C:50  R:53 | 497 |
|  | 19827 | negative regulation of apoptotic process | 0.033  0.032 | C:59  R:57 | 713 |

**Table S12.** Shared gene ontologies present among significantly altered entities in HBEP cells stimulated by the morphological states Filamentous (F) and Reverted (R) compared to unstimulated control cells.

| **Function** | **GO ID** | **GO Term** | **p-value** | **Counts in selection** | **Counts in total** |
| --- | --- | --- | --- | --- | --- |
| Response to abiotic stimulus | 968 | response to hypoxia | 0.000  0.002 | F:18  R:28 | 226 |
| Response to chemical | 8141 | response to organic cyclic compound | 0.047  0.002 | F: 24  R:59 | 670 |
| Immune system process | 1060 | neutrophil homeostasis | 0.020  0.041 | F:3  R:4 | 7 |
|  | 12831 | neutrophil chemotaxis | 0.000  0.036 | F:8  R:9 | 42 |
| Cell death | 4938 | apoptotic process | 0.000  0.000 | F:37  R:79 | 931 |

**Table S13.** Shared gene ontologies present among significantly altered entities in all three morphological states Coliform (C), Filamentous (F) and Reverted (R).

| **Function** | **GO ID** | **GO Term** | **p-value** | **Counts in selection** | **Counts in total** |
| --- | --- | --- | --- | --- | --- |
| Regulation of immune system process | 1948 | positive regulation of leukocyte chemotaxis | 0.004  0.000  0.040 | C:13  F:12  R:11 | 62 |
|  | 5714 | negative regulation of cell proliferation | 0.004  0.000  0.005 | C:54  F:27  R:52 | 585 |
